# Anterior Dislocation of the Elbow-Joint

**Published:** 1867-06

**Authors:** R. P. Hunt

**Affiliations:** Chicago, Ill.


### ANTERIOR DISLOCATION OF THE ELBOW-JOINT.

By R. P. Hunt, M.D., Chicago, Ill.

Early in the war between Mexico and the United States, I was called upon, as the Surgeon of the Second Kentucky Regiment, of which Henry Clay, Jr., was Lieutenant-Colonel, to treat him for an injury caused by the bad action of his horse, an anterior dislocation of the bones of the fore-arm upon the anterior surface of the humerus, and that without fracture of the olecranon process. I was young at the time, and more or less timid; my books in camp were but few, indeed Sir Astley Cooper on *Dislocations and Fractures* formed, at that camp, my entire library on similar affections; in that book I found the accident with which I had to deal entirely ignored, the case, however, existed. The unfractured olecranon and the depression in the rear of the humerus were distinctly felt. There was *no* fracture of the olecranon. I tried to reduce this dislocation by direct traction, without avail; then I flexed the fore-arm upon the arm, changed the extension to near the elbow-joint, repeated the traction, and suddenly, at the proper time, stopped the traction, by extending the fore-arm, the luxation was reduced. A roller bandage, and a dose of morphine to procure ease and sleep, were all that I did. A cold water dressing might possibly have been better, but, be that as it may, Col. Clay was about in a few days, gradually improving the use of his elbow from time to time. He was killed in battle at Buena Vista, gallantly fighting under the flag he had sworn to defend.

Sir Astley Cooper, and all surgeons of his day, even of before, or since, ignore this luxation. For years, I searched the books to find a similar case, without avail. At length, Prof. Gross, in his extensive work upon *Operative Surgery*, recognizes the luxation; he tries to explain it by experiments made upon dead bodies; these experiments may be taken for what they are worth.

Gross says:—Dislocation of the fore-arm *forward*, is an exceedingly rare event, which was formerly supposed to be altogether impossible without previous fraction of the olecranon, or extensive laceration of the soft parts. Modern observation, however, has shown the fallacy of this opinion, by adducing several unequivocal cases in which the displacement existed as a pure, uncomplicated affection. There are two methods of *reduction which may be employed*, one consisting in direct traction or forced flexion, the heel applied as a fulcrum to the lower-third of the arm of the patient under the influence of chloroform. Prof. Frank Hamilton, for whom I have the highest personal and professional esteem, says, "Sir Astley Cooper, Vidal deCassis, and others have denied that this dislocation was possible without fracture of the olecranon process; but Monin, Prior, Velpeau, Canton, and Denucé have each reported one example, so that its existence may now be considered as established."

Prof. Hamilton, I regret to say, in this fact, is wrong in one point at least, he misquotes Vidal (deCassis) in this particular: Vidal says distinctly, that he has reported from different authorities such and such cases; he cannot understand such an accident, but (doubting is not denying) thus it seems that modern surgery has, more or less formally acquiesced in the possibility of anterior ulnar luxations without fracture of the olecranon.

